# Physiological and perceptual effects of two passive back-support exoskeletons during repetitive lifting in healthy adults

**DOI:** 10.1007/s00421-026-06125-9

**Published:** 2026-02-12

**Authors:** Johannes Voß, Jonas Deicke, Ulrich Laufs, Christoph Pökel, Celina Schlosser, Max Schuhte, Maxi Kramer, Olaf Ueberschär, Roberto Falz

**Affiliations:** 1 Institute of Sports Medicine and Prevention, Leipzig University, Rosa-Luxemburg-Str. 20-30, 04103 Leipzig, Germany; 2 Department of Engineering and Industrial Design, Chair of Human-Machine-Interaction, Magdeburg-Stendal University of Applied Sciences, Magdeburg, Germany; 3 Clinic and Polyclinic for Cardiology, University Hospital Leipzig, Leipzig, Germany; 4 Institute for Applied Training Science, Leipzig, Germany

**Keywords:** Wearable technology, Physical strain, Ergonomics, Manual handling, Occupational physical activity

## Abstract

**Purpose:**

Passive back-support exoskeletons (PBEs) are wearable devices designed to reduce strain during physical labor, e.g., repetitive lifting. This study investigated acute effects of two PBEs on cardiac load, energy expenditure, neuromuscular activity, and perceived exertion during repetitive lifting.

**Methods:**

Twenty-six healthy adults performed a standardized lifting task under three conditions: without exoskeleton (FREE), with a rigid PBE (RIGID), and with a soft PBE (SOFT). Cardiac load was assessed via impedance cardiography and blood pressure; energy expenditure via respiratory gas analysis; and neuromuscular activity via surface electromyography. Subjective ratings of exertion and comfort were collected before, during, and after the task. Data were analyzed using repeated measures analyses of variances and mixed-effects models, all followed by post-hoc tests.

**Results:**

Both exoskeletons reduced rate-pressure product (RIGID: −8.1%, *p* = 0.001; SOFT: −6.5%, *p* = 0.003), energy expenditure (RIGID: −13.9%, *p* < 0.0001; SOFT: −9.4%, *p* < 0.0001), and perceived exertion (RIGID: −14.4%, *p* < 0.001; SOFT: −9.5%, *p* = 0.034) compared to FREE. Only RIGID reduced gluteus maximus muscle activation (− 21%, *p* = 0.005). No significant changes were observed in trunk or abdominal muscles. Wearing comfort declined post-task for both devices. No differences occurred between PBEs across all parameters.

**Conclusion:**

This study demonstrates that rigid and soft PBEs consistently reduce physiological and perceptual demands during lifting. By integrating central and peripheral hemodynamic measures, we extend understanding of cardiac unloading. Long-term field studies are needed to assess whether these benefits persist in real-world settings and contribute to musculoskeletal and cardiovascular health.

**Supplementary Information:**

The online version contains supplementary material available at 10.1007/s00421-026-06125-9.

## Introduction

Physical activity is generally associated with positive effects on both physical and mental health (Biddle and Asare 2011; Ciumărnean et al. 2021; Hallal et al. 2006). These benefits, however, primarily apply to leisure-time physical activity and do not necessarily extend to occupational physical activity (OPA). High levels of OPA often result from biomechanical strain caused by repetitive lifting tasks, as commonly seen in logistics, care, military, the energy sector, or construction industries (De Kok et al. 2019). Elevated OPA exposure has been identified as a major contributor to work-related musculoskeletal disorders (WMSDs) (Da Costa and Vieira 2010). These represent the most prevalent work-related health issue in Europe (De Kok et al. 2019). The financial burden of WMSDs is substantial, with estimated costs of approximately 240 billion euros per year, equivalent to about 2% of the gross domestic product of the EU-15 (Bevan 2015). In addition to musculoskeletal outcomes, high OPA is also associated with a 35% increase in risk of severe cardiac events and a 27% higher risk of cardiovascular mortality (Holtermann et al. 2021). One proposed mechanism is the sustained elevation of 24-hour heart rate and blood pressure observed in workers engaged in repetitive or demanding physical tasks – factors known to contribute to cardiovascular risk (Holtermann et al. 2018). Effective preventive strategies are urgently needed to reduce the significant health and socioeconomic burden caused by the consequences of high OPA.

Occupational exoskeletons are wearable assistive devices designed to reduce the physical strain experienced by workers exposed to high levels of OPA (Theurel and Desbrosses 2019). While active exoskeletons use motorized joints powered by external energy sources, passive exoskeletons function without such additional power support. Instead, they assist the person’s movements through passive mechanisms that store and release mechanical energy. Based on their structural components, passive exoskeletons can be further classified into rigid and soft exoskeletons. Recent evidence suggests that the structural design – rigid versus soft – may influence both usability and biomechanical effectiveness (Refai et al. 2024a). The structural design of PBEs may influence how mechanical assistance is distributed across the trunk, hip, and lower limbs. Rigid systems typically redirect external moments through articulated frames, whereas soft designs rely on elastic tension across the torso and thighs. These differing force-transmission mechanisms could elicit distinct physiological responses across cardiac, metabolic, neuromuscular, and perceptual domains, depending on how load is transferred and muscular effort is redistributed. While many exoskeletons are primarily developed to reduce physical strain and prevent WMSDs in occupations with high physical demands, others are designed to augment human capability, for instance, to enable single heavy lifts or to support prolonged isometric postures. These use cases highlight that exoskeletons can serve both assistive and augmentative purposes. The present study focuses on the assistive function of exoskeletons during repetitive lifting tasks, which represent a common and physically demanding component of OPA.

For lifting tasks in particular, passive back-support exoskeletons (PBEs) are intended to reduce static and dynamic demands placed on the posterior chain muscles and the spine. Previous studies have demonstrated that PBEs can reduce physical strain across various laboratory settings typically reducing muscle activity of the trunk or hip extensors by 10–25%, heart rate by 3–6%, energy expenditure by 4–18%, and perceived exertion by 7–22% (Alemi et al. 2020; Luger et al. 2023; Schwartz et al. 2022a; Kermavnar et al. 2021; Ahn et al. 2025; Baltrusch 2020). However, most of these studies have focused on isolated outcome parameters, such as muscle activity, metabolic cost, perceived exertion, or heart rate. A recent meta-analysis emphasized the need for a more multifaceted assessment of physical strain, considering multiple physiological and perceptual domains (Bär et al. 2021).

Biomechanical parameters such as muscle activity are of central importance in understanding the mechanisms of a potential preventive effect of PBEs on WMSDs. Concerning the chronic elevations in resting heart rate and blood pressure observed in workers engaged in physically demanding occupations, cardiac load could be a particularly relevant parameter. Recent findings on the effects of PBEs on cardiac load remain inconsistent (Luger et al. 2023; Schwartz et al. 2022a,^2021^; Bär et al. 2024; Lotz et al. 2009), and to date, these conclusions have relied almost exclusively on heart rate measurements. However, heart rate alone offers only a limited perspective on cardiac load.

Additional hemodynamic parameters, such as rate-pressure product and cardiac output, may provide a more comprehensive understanding of the cardiac response to lifting tasks with and without exoskeleton support. The rate-pressure product is a well-established surrogate measure of myocardial oxygen consumption, and thus a more accurate proxy of cardiac load than heart rate alone (Kitamura et al. 1972). In exercise physiology, thoracic impedance cardiography (ICG) is widely used as a non-invasive and valid method to assess cardiac output under dynamic conditions (Yilmaz et al. 2013; Heydari et al. 2013; Charloux et al. 2000).

The primary aim of this study was to examine the acute physiological and perceptual effects of PBE compared with unassisted lifting. Both a rigid and a soft PBE were included to represent different structural concepts within this device category. We hypothesized that each exoskeleton would reduce cardiac load, energy expenditure, neuromuscular activity, and perceived exertion relative to the unassisted condition. A secondary, exploratory aim was to determine whether the two design types, rigid and soft, differ in the magnitude of these physiological or perceptual effects. By addressing multiple domains of physical strain, this study provides a more comprehensive understanding of the acute impact of PBEs during repetitive lifting.

## Materials and methods

### Participants

Twenty-six healthy adults (13 men, 13 women) participated in this study, and all completed the trial. Table 1 shows the subject characteristics obtained from the preliminary examinations: bioimpedance analysis and cardiopulmonary exercise test. Before participation, an anamnestic health survey was carried out to check for eligibility. To meet the inclusion criteria, participants had to be healthy, between 18 and 35 years of age, and engaged in recreational physical activity at least once per week. None of the participants worked in load-lifting jobs or had prior experience with the explicit lifting task. This study was conducted in accordance with the Declaration of Helsinki, and approved by the Ethics Committee of the Medical Faculty of the University of Leipzig (340/23-ek). All subjects gave written informed consent to their participation.

**Table 1:** Subject characteristics from preliminary examinations

Variable		
Anthropometric data	Mean ± SD	
Age (years)	25.2 ± 3.8	
Height (cm)	175 ± 9.8	
Weight (kg)	71.8 ± 10.4	
BMI (kg/m^2^)	23.3 ± 2.1	
Fat mass (kg)	15.8 ± 4.8	
Lean body mass (kg)	56.4 ± 10.1	
Physical activity volume (h)	6.2 ± 2.1	

Cardiopulmonary exercise test	Baseline	Peak
HR (bpm)	74.9 ± 13.0	187.8 ± 8.5
SV (ml)	89.4 ± 21.0	140.9 ± 28.1
CO (l/min)	6.5 ± 1.2	24.3 ± 4.4
V̇O_2_ (ml/min/kg)	5.5 ± 1.0	47.0 ± 7.3
SBP (mmHg)	125 ± 10	201 ± 21
DBP (mmHg)	75.0 ± 4.7	79.1 ± 3.3
RPE	1.0 ± 0	9.4 ± 0.6

All values are means ± standard deviations

*HR* Heart rate, *SV* Stroke volume, *CO* Cardiac output, *V̇O2* Oxygen uptake, *SBP* Systolic blood pressure, *DBP* Diastolic blood pressure, *RPE* Rate of perceived exertion

### Exoskeletons

Two PBEs were investigated: the soft textile-based Hunic SoftExo Lift V4 (Hunic GmbH, Germany; referred to as SOFT) and the rigid Laevo Flex V3.0 (Laevo B.V., Netherlands; referred to as RIGID). Both devices are back-support exoskeletons designed to reduce lumbar loading. In the Laevo Flex V3.0, force transmission is achieved through hip–thigh interfaces, whereas in the Hunic SoftExo Lift V4, it relies on elastic linkages spanning from the back to the knee and lower leg.

The SOFT PBE consists of shoulder straps, textile cuffs around the knees and ankles, a lightweight back structure made of polyvinyl chloride and carbon, and soft elastic bands. Its mass ranges between 0.95 and 1.15 kg, depending on the size. Elastic elements connect the back structure to the knee cuffs and provide dynamic support through adaptive tension, depending on the movement pattern. The spring stiffness is specified as 1,500 N/m for elongation under 5%, and 175 N/m beyond 5% (Reimeir et al. 2023). Each unit was individually adjusted to the participant’s body dimensions to ensure optimal fit and functionality.

The RIGID PBE features a rigid vest frame, a hip frame, and thigh pads on the anterior aspect of the upper legs. These components are connected via rigid bars and a smart joint system that facilitates torque-assisted trunk and hip extension. Depending on size, the device possesses a mass between 4.0 and 4.4 kg. Assistance is provided by torsion springs located near the greater trochanter, which store mechanical energy during trunk flexion and release it during extension. The exoskeleton was individually fitted for each participant. Actuator strength was standardized across participants at 70% of the maximum setting (medium assistance), delivering a peak support torque of 41 Nm during each lift (van Harmelen and Schnieders 2022).

### Study design

Participants were recruited between October 2023 and March 2024. Each subject completed a two-week examination period consisting of four laboratory visits, each separated by a minimum interval of 48 h to avoid carryover effects. Each session lasted approximately 90 min.

During the first visit, anthropometric data (body weight and height), body composition via bioimpedance analysis (Biacorpus RX4004M, MEDI CAL GmbH, Germany), a resting electrocardiogram (custo cardio BT-A 300, custo GmbH, Germany), and a cardiopulmonary exercise test were obtained.

Visits two, three, and four involved standardized lifting tasks performed under three experimental conditions: (1) without exoskeleton use (FREE), (2) with RIGID, and (3) with SOFT. The order of these conditions was randomized across participants to minimize order effects. Participants were instructed to refrain from strenuous physical activity for at least 24 h before each session.

### Lifting task

**Fig. 1:**
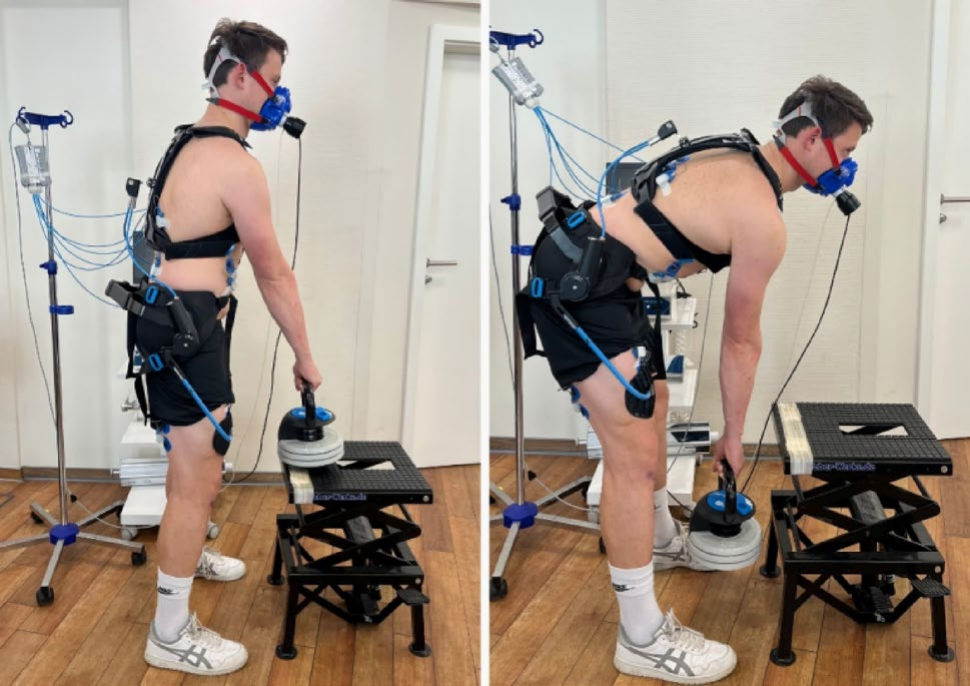
Experimental setup and movement pattern of the repetitive lifting task performed by a male subject wearing the Laevo Flex V3.0. The lifting task was performed using a hip-hinge movement with slight knee flexion

All testing sessions were conducted in a quiet, temperature-controlled laboratory with a target temperature of 20 °C. The lifting platform was individually adjusted to allow subjects to pick up the load with each arm fully extended at hip height, ensuring an ergonomic starting position. Each session began with a 3-minute treadmill warm-up at 8–9 km/h, followed by mobilization exercises for the upper and lower body to support flexibility and motor control. Prior to the lifting task, participants received technique instructions to ensure correct and consistent movement execution. Thereafter, subjects were prepared for surface electromyography (sEMG) application, followed by measurements of maximum voluntary contractions (MVC). Details on the sEMG procedure can be found in the descriptions of data collection below. The lifting task consisted of repeatedly moving a kettlebell with one arm from platform height level to ankle level and back. The kettlebell load corresponded to 15% of each participant’s body mass (mean ± SD: 10.8 ± 2.2 kg). The lifting setup and task sequence are illustrated in Fig. 1. One complete cycle (lifting and lowering) lasted six seconds (2 s down, 2 s up, 2 s rest), paced by acoustic and visual signals to maintain a consistent rhythm. The lifting task was primarily induced through a hip-hinge movement, permitting only minimal knee flexion. Execution of the movement was observationally monitored and applied consistently across all experimental conditions. A sequential depiction of the movement is illustrated in Appendix 1. To reach a physiological steady state, the task was performed continuously for five minutes. To enable blood pressure measurements at the end of each minute and to ensure symmetric loading, participants switched arms every 30 s. During the task, participants were instructed to stand with their feet hip-width apart and their knees slightly flexed.

### Data collection

The main outcome parameters were rate-pressure product (RPP), energy expenditure (EE), neuromuscular activity, rating of perceived exertion (RPE), and wearing comfort.

#### Cardiac load

Heart rate (HR), stroke volume (SV), and cardiac output (CO) were continuously monitored using non-invasive transthoracic ICG (PhysioFlow PF07 Q-Link, Manatec Biomedical, France). Data were collected beat-by-beat and averaged over 10-second intervals. Systolic blood pressure (SBP) was measured manually once per minute using a calibrated aneroid sphygmomanometer (boso BS90, Bosch + Sohn, Germany) on the left upper arm with an adult cuff (24–32 cm). Measurements were taken while the participant held the weight in the right hand, during which the cuff was inflated and gradually deflated throughout the concentric phase of the lifting cycle until the first Korotkoff sound was detected. The participant’s left arm was supported at heart level and stabilized to minimize motion artefacts while remaining relaxed. The corresponding HR for rate–pressure product (RPP) calculation was derived as the mean HR across the last two minutes of the lifting block, matching the time window used for mean SBP determination. Only recordings with a signal quality index ≥ 80% and without visible artefacts were included. RPP was calculated as SBP × HR and averaged across the last two minutes of the lifting block.

#### Energy expenditure

Maximal oxygen uptake (V̇O₂max) was determined for each participant during their first visit using an incremental CPET on an electronically braked semi-recumbent ergometer (Ergoline, Germany). The initial load was set to 30 W with increments of 10 W·min⁻¹ for women and 50 W with increments of 15 W·min⁻¹ for men, continued until volitional exhaustion. V̇O₂max was defined as the highest 30-s average of oxygen uptake achieved. The respiratory gas analyzer (Metalyzer^®^ 3B, Cortex Biophysik GmbH, Germany) was calibrated prior to data collection according to the manufacturer’s guidelines. V̇O₂ and carbon dioxide production (V̇CO₂) were measured breath-by-breath and averaged over 10-second intervals. For each trial, the average V̇O₂ and V̇CO₂ values from the final two minutes were used to represent steady-state metabolic activity. EE was then calculated using the Brockway Equation (Brockway 1987). In line with previous studies (Alemi et al. 2020; Ahn et al. 2025), EE was normalized to the total system mass, i.e., the sum of body mass and exoskeleton mass for each condition (kcal·min⁻¹·kg⁻¹).

#### Perceived exertion and wearing comfort

At baseline and the end of each minute, participants rated their global perceived exertion on the Borg CR scale from 1 (minimum exertion) to 10 (maximum exertion). This allows a minute-by-minute assessment of perceived exertion throughout the task. Participants further rated their wearing comfort on a 100-mm visual analog scale (VAS) before and after the lifting task. During the VAS assessment, participants wore the respective exoskeleton (RIGID or SOFT), and in the FREE condition, no exoskeleton was used. We defined “minimum comfort” as the word anchor for the left end and “maximum comfort” for the right end of the line. We measured the distance (mm) between the “minimum comfort” anchor and the participant’s mark, providing a range of scores from 0 to 100. A higher score indicated higher wearing comfort.

#### Neuromuscular activity

sEMG signals were recorded from seven muscles on the right side of the body: rectus femoris muscle, biceps femoris muscle, gluteus maximus muscle, iliocostalis erector spinae muscle, longissimus erector spinae muscle, rectus abdominis muscle, and external oblique muscle. Skin preparation followed SENIAM recommendations and included shaving, gentle abrasion, and cleaning with 70% isopropyl alcohol (Hermens et al. 2000). Bipolar Ag/AgCl surface electrodes (Kendall™ H34LG) were placed following SENIAM recommendations (Hermens et al. 2000) for rectus femoris muscle, biceps femoris muscle, gluteus maximus muscle, iliocostalis erector spinae muscle, and longissimus erector spinae muscle. For rectus abdominis muscle and external oblique muscle, electrode sites were based on placement guidelines from previous literature (Abdoli-E et al. 2006; Marshall and Murphy 2003). The inter-electrode distance was 20 mm.

sEMG data were recorded using the Ultium EMG system (NORAXON, USA) with a sampling rate of 2000 Hz and processed using the manufacturer’s MR 3.20 software (myoMUSCLE module). A proprietary software-based filter was applied to reduce heart rate artifacts, particularly in iliocostalis erector spinae muscle, longissimus erector spinae muscle, rectus abdominis muscle, and external oblique muscle channels. Signals were band-pass filtered using a Hamming window (10–500 Hz), rectified, and smoothed with a 150-ms root-mean-square algorithm. Root-mean-square values were averaged every 30 s during the lifting task, and the mean of the final two minutes was used for analysis.

The dominance of the participants was not determined, as the function of the exoskeleton does not affect the dominant or non-dominant side differently. For standardization purposes, the sEMG instrumentation was always performed on the right side. During the lifting task, the loads were lifted with the right and left hand in 30-second intervals. The sEMG signals were thus recorded both during the active load (right hand) and during the passive phase (left hand). For the analysis, the mean value was calculated across both phases, so that no separate control or modeling was required for the unloaded side.

To account for interindividual variability, sEMG signals were normalized to the maximum amplitude obtained during isometric maximum voluntary contractions (MVCs). For each muscle, ramp MVCs were performed three times against static resistance, increasing force to maximum within 3–5 s and maintaining it for an equal duration, with 30–60 s rest between attempts. Testing positions followed electromyography guideline recommendations (Hermens et al. 2000). To determine the MVC value, a MVC window was automatically defined using the software to exclude any movement artifacts. The highest value of the processed sEMG data within the time window was taken to normalize the sEMG in the lifting task.

For trunk muscles (rectus abdominis, iliocostalis erector spinae, longissimus erector spinae, external oblique), MVCs were performed using an isokinetic apparatus (Pegasus 3D, Physiomed Elektrotechnik AG, Germany) to immobilize the subjects during the isometric MVCs. Subjects executed an isometric ventral trunk flexion (rectus abdominis muscle), dorsal extension (iliocostalis erector spinae, longissimus erector spinae), and ipsilateral lateral flexion (external oblique muscle). For lower limb MVCs, the rectus femoris muscle was assessed in a seated position (hip and knee flexion of 90°) with free-hanging legs and resistance applied to the distal thigh during leg extension. Biceps femoris muscle was tested in a prone position (knee flexed at 35–45°) with resistance at the distal leg during knee flexion. Gluteus maximus muscle was tested in the same prone position (knee flexed at 90°) with vertical resistance at the midfoot during hip extension.

### Statistical analysis

An a priori power analysis was conducted using G*Power (version 3.1.9.7) (Faul et al. 2007) for a one-way repeated-measures ANOVA with three within-subject conditions (FREE, RIGID, SOFT). Because no prior data were available for our primary cardiac outcome (rate–pressure product), we used oxygen uptake effects from a comparable study (Schwartz et al. 2022a) as a proxy. Assuming a small-to-medium ANOVA effect size (Cohen’s *f* ≈ 0.19), a sample of approximately 30 participants would provide 80% power at α = 0.05, assuming *r* = 0.7 and ε = 1.0. Our final sample of *n* = 26 is therefore close to this target and, together with the within-subject design, provides adequate power to detect medium effects. All data were processed and analyzed using Microsoft Excel 2023 (Microsoft Corporation, USA) and GraphPad Prism 10 (GraphPad Software Inc., USA). Results are reported as mean ± standard deviation (SD). Normality was assessed using the D’Agostino-Pearson test. For normally distributed values, a one-way repeated measures ANOVA was performed with condition (FREE, SOFT, RIGID) as the within-subject factor, followed by Tukey’s post hoc test adjusted for multiple comparisons. Non-normally distributed data were analyzed using the Friedman test with Dunn’s post hoc test. Differences in wearing comfort between the measurement times before and after the lifting task were analyzed using a two-way repeated measures ANOVA (factors: time and condition), followed by Fisher’s least significant difference test. sEMG data were analyzed using a mixed-effects model with restricted maximum likelihood estimation, including “condition” as a fixed effect and “participant” as a random effect. This approach accounts for missing values under the assumption that data are missing at random. Post hoc comparisons were performed using Tukey’s test. Percentage reductions are reported as the arithmetic mean of individual percentage differences between RIGID/SOFT and FREE across participants. For the ANOVA, effect sizes were calculated as Cohen’s *f* and interpreted according to conventional thresholds: *f* = 0.10 (small), *f* = 0.25 (medium), and *f* = 0.40 (large) (Cohen 1992). For the Friedman test, Kendall’s W was reported as an effect size and classified as follows: *W* = 0.10 (small), *W* = 0.30 (medium), and *W* = 0.50 (large) (Cohen 1992). The magnitude of pairwise differences was quantified using Cohen’s d, with values interpreted as *d* = 0.20 (small), *d* = 0.50 (medium), and *d* = 0.80 (large) (Cohen 1992). Statistical significance was defined as *p* < 0.05 for all analyses.

## Results

**Fig. 2:**
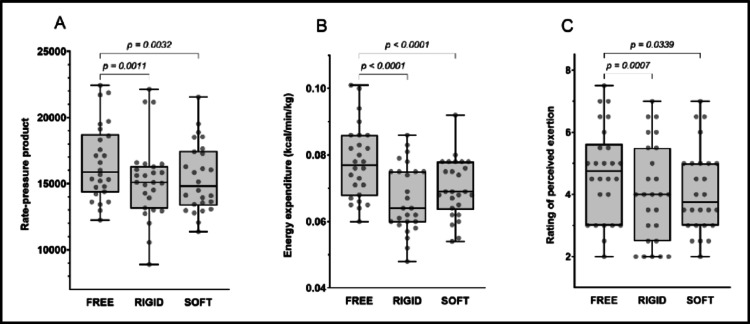
Boxplots show the median (horizontal line), interquartile range (box), and whiskers (1.5 × IQR). Individual data points (n = 26) are overlaid to illustrate within-subject variability. **A **Rate–pressure product, **B** energy expenditure, and **C** rating of perceived exertion during repetitive lifting under three conditions: FREE (no exoskeleton), RIGID (rigid passive back-support exoskeleton), and SOFT (soft passive back-support exoskeleton). Significant pairwise differences between conditions are indicated by the reported p-values from post-hoc analysis

Both passive exoskeletons significantly reduced physiological and perceptual load compared to the unassisted condition (FREE). Specifically, exoskeletons reduced RPP (RIGID: −8.1%; SOFT: −6.5%; Fig. 2), EE (RIGID: −13.9%; SOFT: −9.4%; Fig. 2), and RPE (RIGID: −14.4%; SOFT: −9.5%; Fig. 2).

Regarding neuromuscular activity, only RIGID led to a significant reduction in gluteus maximus activation (− 21% vs. FREE; *p* = 0.004), while SOFT did not produce significant changes in any measured muscle group.

Analyzing wearing comfort with a two-way repeated measures ANOVA revealed a significant time effect (*p* = 0.001), no exoskeleton effect (*p* = 0.739), and no interaction effect (*p* = 0.986). Post-hoc analysis indicated a significant decrease in wearing comfort from pre- to post-lifting task for both RIGID (− 11.6%; pre: 67.4 ± 18.9, post: 59.6 ± 22.8; MD = − 7.8 [95% CI − 15.1, − 0.5]; *p* = 0.033) and SOFT (− 11.2%; pre: 69.2 ± 21.3, post: 61.5 ± 22.5; MD = − 7.7 [95% CI − 15.0, − 0.5]; *p* = 0.036).

No statistically significant differences were observed between RIGID and SOFT or between baseline values across any of the measured parameters. Table 2 shows a complete overview of our results.

**Table 2:** Effects of passive back-support exoskeletons during a repetitive lifting task

Variable	FREE (*n* = 26)	RIGID (*n* = 26)	SOFT (*n* = 26)	Main model *p*-value; effect size	RIGID − FREE MD [95% CI]; *p*-value; effect size	SOFT − FREE MD [95% CI]; *p*-value; effect size
Cardiac load						
HR (bpm)	111.2 ± 15.9	104.5 ± 16.5	106.4 ± 15.4	***p*** **< 0.001**^a^; *W* = 0.404	−6.6 [− 10.6, − 2.7]; ***p*** **< 0.001**; *d* = − 0.829	−4.8 [− 9.4, 0.2]; ***p*** **= 0.038**; *d* = − 0.523
SBP (mmHg)	149 ± 11	145 ± 12	145 ± 12	***p*** **= 0.044**^b^; *f* = 0.164	−3.9 [− 8.4, 0.6]; *p* = 0.094; *d* = − 0.437	−3.8 [− 7.2, − 0.5]; ***p*** **= 0.023**; *d* = − 0.568
RPP (x 10^3^)	16.6 ± 2.9	15.2 ± 3.0	15.4 ± 2.6	***p*** **= 0.002**^b^; *f* = 0.218	−1.4 [− 2.2, − 0.6]; ***p*** **= 0.001**; *d* = − 0.817	−3.9 [− 8.4, − 0.6]; ***p*** **= 0.003**; *d* = − 0.733
SV (ml)	66.5 ± 15.3	68.1 ± 16.8	67.9 ± 12.5	*p* = 0.630^b^; *f* = 0.048	1.6 [− 3.7, 6.9]; *p* = 0.741; *d* = − 0.148	1.4 [− 3.2, 5.9]; *p* = 0.743; *d* = − 0.148
CO (l/min)	7.32 ± 1.72	7.07 ± 2.04	7.13 ± 1.15	*p* = 0.191^a^; *W* = 0.092	−0.25 [− 0.89, 0.4]; *p* = 0.214; *d* = − 0.178	−0.19 [− 0.78, 0.4]; *p* > 0.999; *d* = − 0.152
Metabolic cost						
V̇O_ 2_ (l/min)	1.16 ± 0.28	1.06 ± 0.26	1.05 ± 0.22			
V̇O_ 2_ (ml/min/kg)	16.0 ± 2.3	14.6 ± 2.1	14.6 ± 1.8			
%V̇O_2_max (%)	34.7 ± 6.2	31.6 ± 5.4	31.7 ± 5.5			
V̇CO_2_ (l/min)	0.95 ± 0.22	0.87 ± 0.22	0.88 ± 0.20			
RER	0.82 ± 0.04	0.83 ± 0.05	0.83 ± 0.05			
EE (kcal/min)	5.62 ± 1.33	5.13 ± 1.26	5.12 ± 1.07			
EE (kcal/min/kg)	0.078 ± 0.011	0.067 ± 0.010	0.070 ± 0.009	*p* **< 0.0001**^b^; *f* = 0.464	−0.011 [− 0.014, − 0.008]; *p* **< 0.0001**; *d* = − 1.813	−0.008 [− 0.011, − 0.004]; *p* **< 0.0001**; *d* = − 1.143
Perceived exertion						
RPE						
1 min	2.0 ± 0.6	1.9 ± 0.8	1.8 ± 0.6			
2 min	2.8 ± 1.0	2.7 ± 1.0	2.5 ± 1.0			
3 min	3.8 ± 1.4	3.4 ± 1.1	3.3 ± 1.1			
4 min	4.7 ± 1.6	4.0 ± 1.4	3.9 ± 1.4			
5 min	4.7 ± 1.6	4.1 ± 1.8	4.4 ± 1.5			
Ø 4–5 min	4.7 ± 1.5	4.0 ± 1.6	4.1 ± 1.4	***p*** **= 0.004**^b^; *f* = 0.201	−0.7 [− 1.0, − 0.3]; ***p*** **< 0.001;** *d* = − 0.850	−0.6 [− 1.1, − 0.0]; ***p*** **= 0.034;** *d* = − 0.534
Neuromuscular activity						
Biceps femoris muscle (%)	11.7 ± 4.1	9.1 ± 3.7	11.2 ± 4.8	***p*** **= 0.028**^c^	−2.4 [− 5.0, 0.1]; *p* = 0.058; *d* = − 0.496 (25)	−0.8 [− 3.1, 1.5]; *p* = 0.655; *d* = − 0.472 (25)
Gluteus maximus muscle (%)	9.4 ± 3.4	7.3 ± 3.4	8.2 ± 4.0	***p*** **= 0.002**^c^	−2.2 [− 3.8, − 0.6]; ***p*** **= 0.005**; *d* = − 0.771 (22)	−1.4 [− 2.8, 0.1]; *p* = 0.060; *d* = − 0.554 (23)
Iliocostalis erector spinae muscle (%)	21.3 ± 8.4	18.1 ± 6.2	19.7 ± 4.8	***p*** *= *0.144^c^	−3.6 [− 8.5, 1.3]; *p* = 0.177; *d* = − 0.377 (24)	−1.2 [− 5.5, 3.0]; *p* = 0.752; *d* = − 0.396 (24)
Longissimus erector spinae muscle (%)	23.0 ± 8.8	19.9 ± 5.3	20.0 ± 7.4	***p*** *= *0.124^c^	−3.5 [− 8.1, 1.2]; *p* = 0.178; *d* = − 0.385 (24)	−3.1 [− 8.0, 1.7]; *p* = 0.258; *d* = − 0.260 (24)
Rectus femoris muscle (%)	3.0 ± 1.5	2.8 ± 1.9	2.3 ± 1.5	***p*** *= *0.129^c^	−0.3 [− 1.3, 0.7]; *p* = 0.783; *d* = − 0.140 (24)	−0.8 [− 1.6, 0.0]; *p* = 0.056; *d* = − 0.499 (25)
Rectus abdominis muscle (%)	2.6 ± 2.0	2.4 ± 2.1	2.5 ± 1.8	***p*** *= *0.807^c^	−0.2 [− 1.4, 1.0]; *p* = 0.897; *d* = − 0.093 (24)	−0.3 [− 1.3, 0.7]; *p* = 0.696; *d* = − 0.145 (24)
External oblique muscle (%)	4.2 ± 1.7	4.6 ± 1.9	3.5 ± 2.0	***p*** *= *0.054^c^	0.3 [− 0.8, 1.5]; *p* = 0.770; *d* = 0.142 (25)	−0.9 [− 1.8, 0.0]; *p* = 0.070; *d* = − 0.388 (24)

All values represent the average of the last two minutes of the lifting task except for RPE values of minutes 1–5

Values are presented as means ± SDs or as mean differences (MD) between conditions with 95% confidence intervals (CI). For variables analyzed with Friedman tests, MD [95% CI] are reported descriptively from the raw data

Effect sizes: *W* = Kendalls *W*; *f* = Cohen’s *f*; *d* = Cohen’s d

Significant results are in bold font

*HR* heart rate, *SV* stroke volume, *CO*: cardiac output, *SBP* systolic blood pressure, *RPP* rate-pressure product, *V̇O*_*2*_ oxygen uptake, *%V̇O*_*2*_*max* oxygen uptake relative to VO_2_max, *V̇CO*_*2*_ carbon dioxide production, *EE* energy expenditure, *RPE* rating of perceived exertion, *sEMG* surface electromyography

Due to missing sEMG data, the number of data sets varies across muscles. This is indicated by (n) following the p-values and effect sizes

Symbols indicating the statistical analyses: Friedman test (^a^); one-way repeated measures ANOVA (^b^); mixed-effects model (^c^)

## Discussion

This study examined the acute effects of two PBEs on multiple domains of physical strain during a standardized five-minute repetitive lifting task. The primary comparison examined exoskeleton-assisted versus unassisted lifting, using one rigid and one soft PBE to represent distinct structural concepts within this category. Both exoskeletons reduced physiological and perceptual strain in healthy adults. By incorporating blood pressure measurements and ICG, this study is, to our knowledge, the first to comprehensively assess cardiac load during dynamic lifting with PBEs.

### Cardiac load

Both PBEs reduced cardiac load compared with the unassisted condition. HR decreased by 5.9% with the RIGID PBE and by 4.0% with the SOFT PBE relative to the unassisted condition. These effects correspond to moderate-to-large magnitudes (*d* = − 0.52 to − 0.83), suggesting consistent physiological unloading across participants. Our findings fall within the upper range of previous laboratory studies, which have mostly reported small or non-significant HR reductions during repetitive lifting with PBEs (Erezuma et al. 2022; Schwartz et al. 2022b; Schmalz et al. 2022; Luger et al. 2023). For instance, Luger et al. (2023) observed a − 1.5 bpm reduction during industrial tasks, and Erezuma et al. (2022) reported a non-significant − 4 bpm decrease under comparable lifting conditions. The slightly stronger effects in the present study may be attributed to the continuous, five-minute lifting protocol with sustained trunk flexion, which elicits a more stable cardiovascular steady state and allows small differences in mechanical assistance to manifest physiologically. In contrast, short-duration or mixed-task protocols often induce high inter-trial variability, masking subtle HR changes. Field investigations have yielded divergent results, with some showing increased HR despite mechanical unloading (Bennett et al. 2023; Marino 2019), possibly due to movement restriction or discomfort as reported by Marino et al. (2019). This underscores that task context and device fit can substantially influence cardiovascular responses to exoskeleton use.

Heart rate alone does not adequately capture cardiac load, as it excludes key hemodynamic factors such as peripheral resistance (Gobel et al. 1978). To address this, we assessed PBE use with ICG and concurrent blood pressure monitoring during repetitive lifting, enabling calculation of RPP as a surrogate for myocardial oxygen demand (Gobel et al. 1978; Sembulingam and Ilango 2015). SBP decreased significantly by 2.5% with SOFT, while a similar reduction with RIGID did not reach significance. Although the observed reductions in SBP were small, even modest decreases in SBP have been shown to lower cardiovascular risk when sustained over time (Canoy et al. 2022). In this context, the acute reductions observed here may still be physiologically relevant for repetitive lifting tasks. Importantly, our findings reflect acute physiological responses during a short, standardized laboratory task, and any implications for chronic or occupational cardiovascular risk would require confirmation in longitudinal field studies.

Both devices reduced RPP compared with the unassisted condition, indicating lower acute cardiac strain. The decrease was slightly but non-significantly greater with the RIGID (− 8.1%) than with the SOFT device (− 6.5%). The reductions in HR and RPP occurred alongside stable ICG-derived SV and with only modest decreases in SBP, indicating that the observed cardiac unloading was primarily chronotropic, with a minor contribution from reduced afterload. Notably, these values reflect an acute 5-minute task in young, active adults and should not be equated with chronic cardiovascular risk. RPP remains a useful surrogate of myocardial oxygen demand for between-condition comparisons, but its clinical interpretation depends on exposure duration and cumulative workload. Therefore, the present reductions indicate lower instantaneous cardiac strain, yet do not imply a change in risk classification under occupational conditions without evidence over longer time frames. Although RPP values remained within published “risk-zone” ranges (Sarnoff et al. 1957; Fletcher et al. 1979), this contextual benchmark is not designed for short, standardized laboratory tasks, and should be interpreted cautiously here. Overall, both PBEs effectively reduced acute cardiac load compared with unassisted lifting, indicating lower instantaneous cardiac strain during repetitive lifting under controlled laboratory conditions.

### Energy expenditure

Both exoskeletons significantly reduced EE during the lifting task compared with the unassisted condition. The reduction averaged 13.9% with the RIGID and 9.4% with the SOFT device, both representing large effects. These values fall within the upper range of previous laboratory studies, which have reported EE decreases between approximately 5% and 18% (Ahn et al. 2025; Alemi et al. 2020, 2022; Erezuma et al. 2022; Schmalz et al. 2022; Baltrusch et al. 2019, 2020; Schwartz et al. 2022a). Previous studies indicate that task complexity moderates the metabolic benefits of PBEs. The largest reductions in energy expenditure are typically observed in standardized, sagittal-plane lifting tasks (Ahn et al. 2025; Baltrusch et al. 2019), whereas asymmetric or multi-directional tasks yield smaller effects (Alemi et al. 2020). This pattern is biomechanically plausible, as the assistance provided by passive devices is most effective when joint torques act predominantly in flexion–extension. Increased trunk stabilization demands and altered movement strategies during complex tasks may further attenuate the unloading potential. The lifting task in the present study was standardized as a repetitive hip-hinge movement, representing a common but mechanically favorable lifting technique for exoskeleton assistance. Under such optimized conditions, the device support aligns closely with hip extension moments, maximizing support efficiency and likely contributing to the magnitude of the observed metabolic savings. In real-world settings with more variable movement patterns, postural asymmetries, or load dynamics, these effects may therefore be smaller or less consistent.

The RIGID device meaningfully unloaded the gluteus maximus (muscle activation − 21% vs. FREE, *d* = − 0.77), likely representing a contributing factor to the observed metabolic savings during hip-dominant lifting. However, as no other measured muscle groups showed statistically significant reductions, this suggests that the substantial reductions in whole-body EE (− 13.9% vs. FREE, *d* = − 1.81) may reflect unmeasured mechanisms, such as lower co-contraction, reduced stabilizing demands, or subtle distributed unloading beyond the muscles captured by sEMG. The SOFT device may reduce EE by distributing assistance across both the hip and knee joints. This mechanism may spread unloading across a broader set of muscle groups, resulting in smaller local sEMG changes while still lowering whole-body EE (− 9.4% vs. FREE, *d* = − 1.14). This dissociation between localized sEMG findings and reductions in EE is consistent with the interpretation that EE reflects the global metabolic cost of the task, including contributions from muscles not captured by sEMG, and that unmeasured, distributed mechanisms likely contributed to the observed metabolic savings.

The slightly greater reductions in EE and cardiac indices observed with the RIGID PBE likely reflect its distinct mechanical principle. The RIGID PBE transmits torque through gas-spring linkages spanning the trunk–hip axis, directly supporting hip and spinal extension, whereas the SOFT PBE distributes elastic tension from the back to the knees and ankles, providing more compliant and dynamic assistance that also involves the lower limbs. As the lifting technique was standardized as a hip-hinge pattern with limited knee flexion, individual variations toward more leg-dominant lifting techniques could modulate these biomechanical and physiological effects in occupational settings.

Mean relative oxygen uptake during lifting corresponded to approximately 31–35%V̇O₂max, clearly within the moderate intensity domain as defined by Poole and Jones (2012), i.e., below the critical power and well within a sustainable metabolic steady state. Within this range, even small reductions in V̇O₂ represent a meaningful gain in aerobic reserve below the fatigue threshold. The moderate decreases (3% points) in metabolic demand observed with both exoskeletons could enhance sustainability and delay fatigue across repeated or prolonged lifting sequences. However, the persistence of this effect likely depends on task variability, load-handling dynamics, and compensatory adjustments in real-world conditions where movement and load demands are less controlled than in laboratory settings. The slightly greater reductions in EE (− 13.9%) and RPP (− 8.1%) with the RIGID device further support the notion that concentrated hip-extensor unloading yields measurable systemic relief.

As our study followed a within-subject design, each participant served as their own control, minimizing potential bias from sex-related differences in absolute cardiovascular or metabolic levels. Nevertheless, biological sex can modulate hemodynamic regulation and oxygen transport capacity (Coe and Astorino 2024; Diaz-Canestro et al. 2022; Del-Cuerpo et al. 2023), and future studies with larger samples may examine whether the magnitude of exoskeleton-induced unloading differs between sexes.

### Neuromuscular activity

Both PBEs showed numerically lower sEMG amplitudes in selected muscles. However, statistically significant neuromuscular unloading was observed only for the RIGID PBE. The most pronounced effect was observed in the gluteus maximus, where activity decreased by 21% with the RIGID and by 14% with the SOFT PBE. While the reduction reached statistical significance only for the RIGID PBE, the non-significant changes observed with the SOFT device do not constitute confirmatory evidence of neuromuscular unloading.

Although stoop lifting induces high lumbar extensor demand, the RIGID PBE primarily generated external hip-extension torque. This selectively unloaded the gluteus maximus while leaving spinal extensor activation unchanged. Across studies, the most consistent reductions are reported for the spinal erectors, particularly under static or deep forward-bending tasks, with decreases between 10% and 40% (Alemi et al. 2019; Baltrusch et al. 2020; Bosch et al. 2016; Di Natali et al. 2024). Under more dynamic lifting conditions with moderate trunk flexion angles (25–40°), as reported by Ahn et al. (2025) and Eskandari et al. (2024), reductions in spinal erector activity tend to diminish or become statistically non-significant, suggesting that unloading magnitude depends on both trunk angle and movement stability. Reductions in gluteus maximus activation are typically smaller and more variable, consistent with our findings, and appear to depend on whether the device provides direct torque assistance at the hip. To our knowledge, no prior study has evaluated a textile-based PBE with anchoring below the knee. The SOFT PBE therefore represents a mechanically distinct configuration that could, in principle, partially unload the rectus femoris through its extended hip–knee–ankle force path. In our data, this unloading trend did not reach statistical significance (*p* = 0.06, *d* = − 0.50). A plausible explanation is that the compliant textile structure may produce lower peak stiffness and temporally dispersed assistance, causing a portion of the transmitted force to dissipate along the distal segments rather than generating concentrated unloading at the thigh. This pattern aligns with previous evaluations of thigh-anchored textile PBEs, which typically reported less muscular unloading than rigid PBEs (Refai et al. 2024b; Schwartz et al. 2021; Alemi et al. 2019). These contrasted effects between the two PBEs likely reflect their distinct load transmission mechanisms. The RIGID PBE redirects torque across the trunk–hip axis via gas-spring linkages, producing concentrated hip extension support, whereas the SOFT PBE provides distributed, compliant assistance with smaller local unloading effects. Accordingly, reductions in gluteus maximus activation were statistically evident only with the RIGID PBE, whereas the SOFT PBE exhibited smaller, non-significant decreases in both gluteus maximus and biceps femoris, consistent with its extended load path toward the lower leg.

Neuromuscular activity in the spinal-extensor muscles and abdominal muscles did not change significantly. However, rectus abdominis and external oblique activity varied across conditions, showing increases under the RIGID and variable responses under the SOFT PBE. Prior studies have similarly reported heterogeneous effects on trunk musculature, possibly due to differences in lift height, movement strategy, and exoskeletal force transmission (Kermavnar et al. 2021; Alemi et al. 2019).

Taken together, the original neuromuscular hypothesis was only partially supported, as statistically significant unloading was confirmed for the RIGID but not for the SOFT PBE. Importantly, the assessed outcomes capture different levels of physiological strain. While sEMG reflects localized muscle activity, EE and cardiac load reflect the global cost of the task. The consistent reductions in EE and cardiac load therefore indicate a reduction in overall muscular demand that is likely distributed across multiple muscle groups, resulting in modest and often non-significant changes at the level of individual muscles assessed via sEMG.

### Subjective perception

Both exoskeletons significantly reduced perceived exertion during repetitive lifting (RIGID: −14.4%; SOFT: −9.5% vs. FREE), consistent with previous findings (Alemi et al. 2020; Ahn et al. 2025; Lotz et al. 2009; Madinei et al. 2020). Alemi et al. (2020) reported a greater reduction (− 31%) when assessing localized lower-back exertion, which may explain the discrepancy to our general RPE measurement. Despite reductions in perceived exertion, both devices were associated with a significant decline in wearing comfort post-task. This aligns with prior research highlighting localized discomfort, particularly in the chest, waist, and thighs (Alemi et al. 2020; Erezuma et al. 2022; Madinei et al. 2020). In terms of subjective discomfort, prior studies have reported low to moderate (Madinei et al. 2020) and moderate to high (Alemi et al. 2020) discomfort levels during the use of PBEs. Our findings support the notion that, while PBEs may reduce physiological and perceptual strain, they may also introduce new sources of discomfort, likely due to mechanical interface pressure or movement restrictions. This trade-off between support and comfort is critical, as user acceptance and long-term adherence will ultimately determine the effectiveness of exoskeletons in occupational settings (Siedl and Mara 2021; Riemer and Wischniewski 2023). In summary, PBEs appear to reduce perceived exertion during repetitive lifting, which may contribute to improved task tolerance. However, this benefit must be weighed against potential declines in wearing comfort.

### Limitations

The standardized, symmetric lifting task with moderate load (15% of body weight) provided a controlled setting for physiological measurement. However, this single-task design does not reflect the task variability typical of physically demanding occupations, where workers regularly have to switch between lifting, carrying, reaching, and asymmetric handling. In such contexts, exoskeletons may support certain tasks while being neutral or even detrimental to others, for example due to added mass or reduced mobility. Likewise, the sample consisted of young, healthy, and physically active adults, which may limit generalizability to older or less fit workers typically exposed to high physical workloads (Holtermann et al. 2021). Although both men and women were included, the study was not powered to detect sex-specific effects. Biological sex may therefore represent a source of interindividual variability in hemodynamic and metabolic responses that should be addressed in future research. Moreover, the study examined only acute responses, and it remains unclear whether the observed reductions in strain translate into long-term musculoskeletal or cardiovascular benefits under field conditions. Additional methodological constraints should also be acknowledged. Although assistance levels were standardized (e.g., Laevo Flex V3.0 at 70%), individualized adjustments might have influenced comfort and mechanical effectiveness. Because outcomes were analyzed as absolute steady-state work values rather than baseline-normalized differences, the results reflect between-condition differences at comparable workload levels rather than deviations from individual rest. As baseline values did not differ between conditions, this approach is unlikely to have affected the magnitude or direction of the reported effects. Finally, limitations apply to the sEMG data. Despite careful electrode placement, mechanical interference or minor electrode displacement cannot be fully excluded. Due to signal loss or artifacts, sEMG recordings from several muscles had to be discarded, resulting in reduced sample sizes and potentially limited statistical power to detect small-to-moderate effects. In addition, the sEMG setup focused on selected superficial muscles and did not capture stabilizing musculature or co-contraction patterns, which may contribute to whole-body EE. These constraints likely limited the sensitivity of sEMG to detect distributed neuromuscular unloading and may help explain the observed discrepancy between localized sEMG findings and systemic metabolic outcomes. Furthermore, only the right side of the body was measured, precluding bilateral comparisons. Future studies should therefore include bilateral recordings, account for handedness, and apply normalization procedures that better reflect dynamic muscle activity during lifting.

## Conclusions

PBEs reduced acute cardiovascular, metabolic, and perceptual effort compared with unassisted lifting. Using blood pressure measurements and ICG, this study provides a more comprehensive assessment of cardiac load, capturing both central and peripheral cardiovascular responses. Despite heterogeneous muscular effects across regions, both exoskeletons lowered overall metabolic and cardiac demands, indicating systemic physiological relief. Testing two structurally distinct PBEs under identical conditions increases the generalizability of the results across exoskeleton types and suggests that unloading effects are a consistent feature of PBEs. While PBEs effectively reduced perceived effort, decreased comfort points to the need for balancing mechanical support with ergonomic integration to ensure user acceptance. Future laboratory studies should examine how unloading effects vary with task duration and load intensity to optimize exoskeleton use. Based on these insights, long-term field studies are needed to assess whether the observed acute benefits persist in occupational environments and contribute to musculoskeletal and cardiovascular health.

## Supplementary Information

Below is the link to the electronic supplementary material.

Supplementary Material 1

## Data Availability

De-identified datasets are available from the corresponding author upon reasonable request under a controlled data-use agreement.

## Abbreviations

ANOVA: Analysis of variance
CO: Cardiac output
DBP: Diastolic blood pressure
HR: Heart rate
ICG: Impedance cardiography
MVC: Maximal voluntary contraction
OPA: Occupational physical activity
PBE: Passive back-support exoskeleton
RPE: Rating of perceived exertion
RPP: Rate-pressure product
SBP: Systolic blood pressure
SD: Standard deviation
sEMG: Surface electromyography
SV: Stroke volume
VAS: Visual analog scale
V̇CO₂: Carbon dioxide production
V̇O₂: Oxygen uptake
%V̇O₂max: Percentage of maximal oxygen uptake
WMSD: Work-related musculoskeletal disorder
