# Supplementary material for: Physiological and perceptual effects of two passive back-support exoskeletons during repetitive lifting in healthy adults
Source: Eur J Appl Physiol. 2026 Feb 12;126(6):3289–302. doi: 10.1007/s00421-026-06125-9 (PMC13287259; doi:10.1007/s00421-026-06125-9)
Supplement: Supplementary file 1 — Supplementary Material 1 [file 421_2026_6125_MOESM1_ESM.docx]

**Supplementary material – Depiction of the lifting task**

**
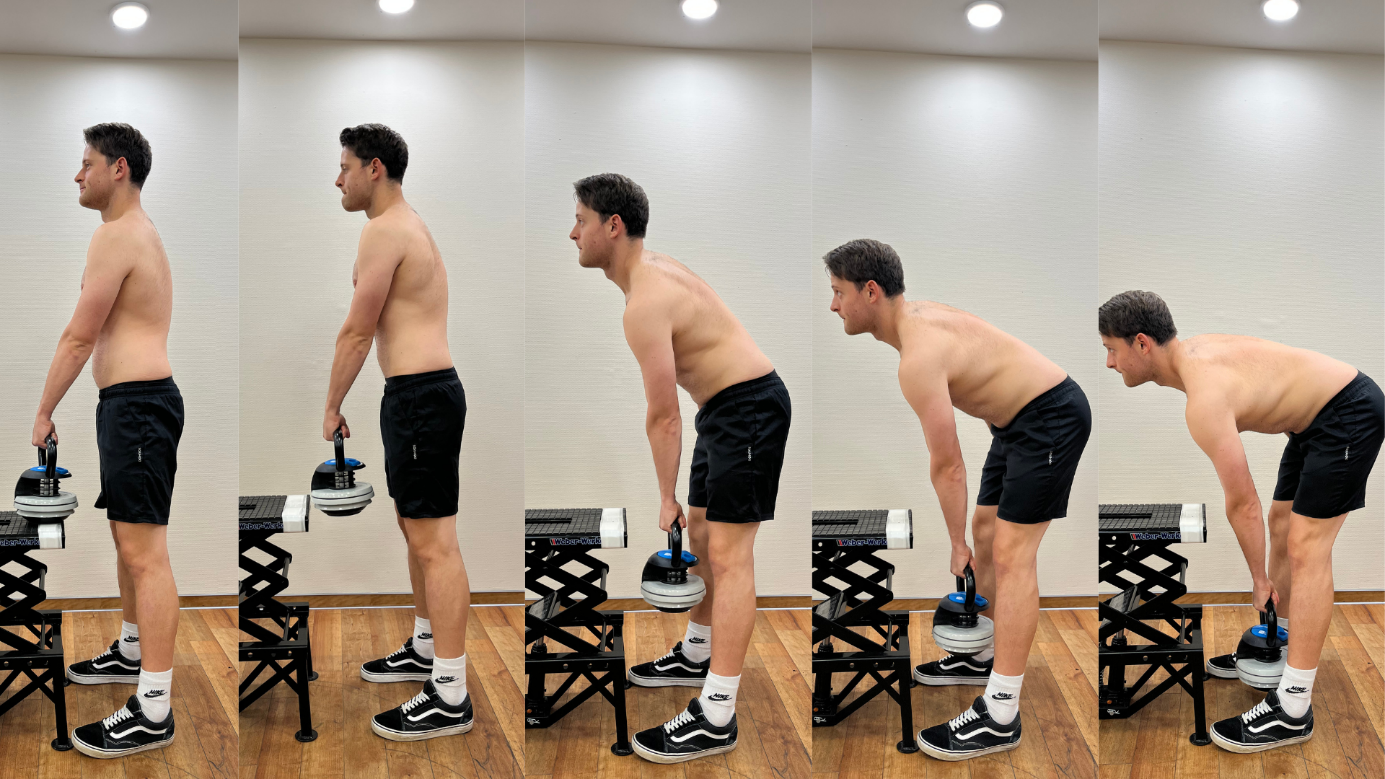
**

**Fig 1** The subject moves the load downwards.

**
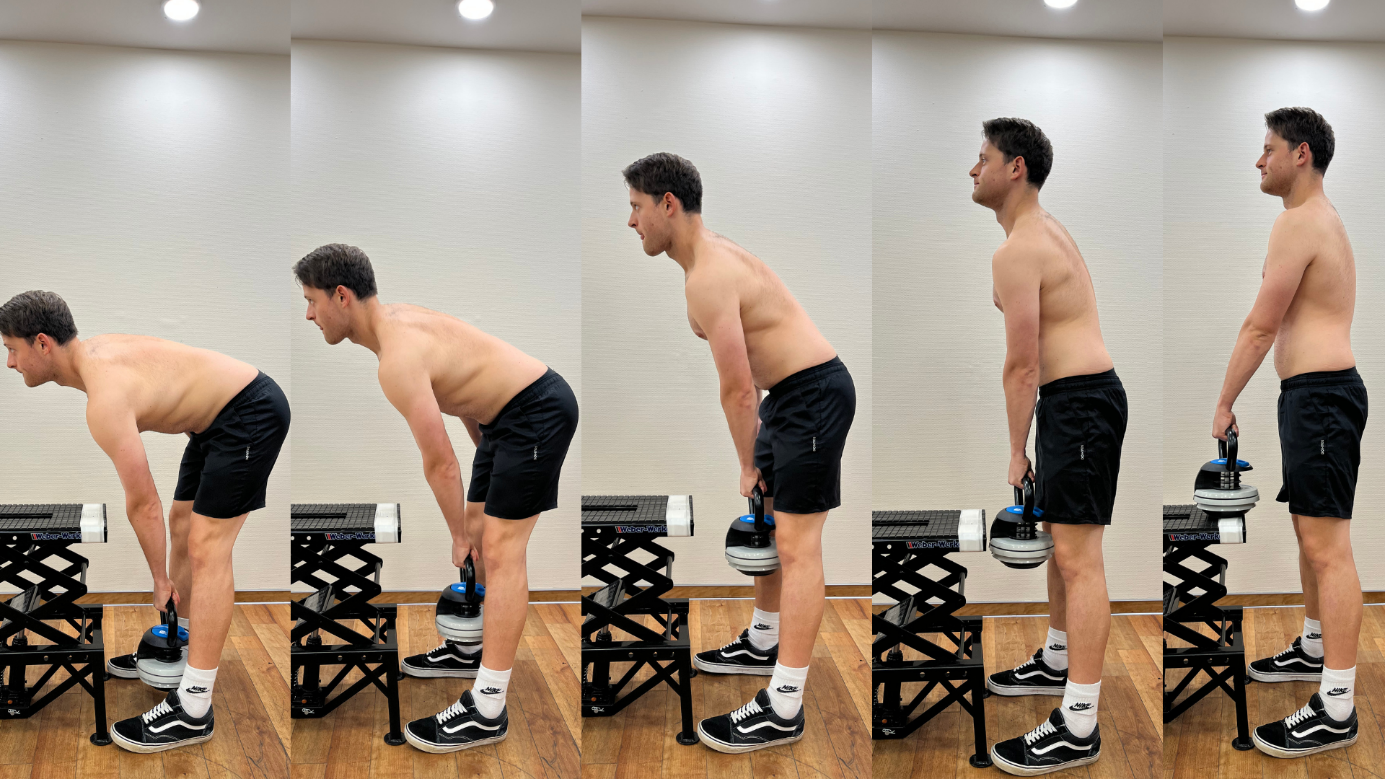
**

**Fig 2** The subject moves the load upwards.
